# Managing hypertension in rural Gambia and Kenya: Protocol for a qualitative study exploring the experiences of patients, health care workers, and decision-makers

**DOI:** 10.3310/nihropenres.13523.2

**Published:** 2024-04-16

**Authors:** Brahima A. Diallo, Syreen Hassan, Nancy Kagwanja, Robinson Oyando, Jainaba Badjie, Noni Mumba, Andrew M. Prentice, Pablo Perel, Anthony Etyang, Ellen Nolte, Benjamin Tsofa, IHCoR-Africa Collaborators

**Affiliations:** 1 Nutrition and Planetary Health, MRC Unit The Gambia at LSHTM, Banjul, Other / None, 273, The Gambia; 2 London School of Hygiene and Tropical Medicine, London, UK; 3 KEMRI Wellcome Trust Research Programme, Kilifi, Kenya

**Keywords:** Hypertension, intervention, community-centred, management, control, health care workers, context

## Abstract

**Background:**

Hypertension is the single leading risk factor for premature death in Sub-Saharan Africa (SSA). Prevalence is high, but awareness, treatment, and control are low. Community-centred interventions show promise for effective hypertension management, but embedding such interventions sustainably requires a good understanding of the wider context within which they are being introduced. This study aims to conduct a systematic health system assessment exploring the micro (patients/carers), meso (health care workers and facilities), and macro (broader system) contexts in rural Gambia and Kenya.

**Methods:**

This study will utilise various qualitative approaches. We will conduct (i) focus group discussions with people living with hypertensive to map a ‘typical’ patient journey through health systems, and (ii) in-depth interviews with patients and family carers, health care workers, decision-makers, and NCD partners to explore their experiences of managing hypertension and assess the capacity and readiness of the health systems to strengthen hypertension management. We will also review national guidelines and policy documents to map the organisation of services and guidance on hypertension management. We will use thematic analysis to analyse data, guided by the cumulative complexity model, and theories of organisational readiness and dissemination of innovations.

**Expected findings:**

This study will describe the current context for the management of hypertension from the perspective of those involved in seeking (patients), delivering (health care workers) and overseeing (decision-makers) health services in rural Gambia and Kenya. It will juxtapose what should be happening according to health system guidance and what is happening in practice, drawing on the experiences of study participants. It will outline the various barriers to and facilitators of hypertension management, as perceived by patients, providers, and decision-makers, and the conditions that would need to be in place for effective and sustainable implementation of a community-centred intervention to improve the management of hypertension in rural settings.

## Introduction

Hypertension is a major cause of premature mortality, accounting for an estimated 10.7 million deaths globally ^1^. Sub-Saharan Africa (SSA) has the highest burdens, with prevalence rates at around 30% ^2^, and in this region, hypertension was implicated in about 1 million deaths in 2019, double the number in 1990 ^3^.

The high burden of hypertension poses significant challenges to health systems in SSA where a high proportion of the population with hypertension remains unaware, undiagnosed, and inadequately or not treated ^4, 5^. It has been estimated that 73% of people with hypertension in SSA do not know about their hypertensive status, 82% of those with hypertension are not receiving treatment, and 93% do not have their blood pressure controlled ^5^. Poorly controlled hypertension is associated with high levels of disability, productivity losses and major socioeconomic costs, exacerbating poverty and increasing health inequalities ^6, 7^. This is a particular concern for rural areas, where health systems are generally weak and case-fatality for cardiovascular diseases is high ^8^.

There is an urgent need to reduce the hypertension-related burden in SSA through effective control along the care pathway; from early detection to timely effective management ^9^. However, there are multiple and complex barriers to achieving this. At the individual level, these include the asymptomatic nature of hypertension, lack of understanding of its potentially serious consequences and competing priorities around home and work, making people only seek care when they experience complications ^10– 14^. Such barriers are compounded by misconceptions about aetiology and the potential benefits of pharmacotherapy ^13^, which, along with an inability to afford medication, can lead to poor treatment adherence. At the health care worker level, poor communication, lack of skills and competencies, limited number of trained health workers, and inadequate referral systems complicate optimal treatment ^15– 20^. System-level barriers include poor access to health facilities, particularly in rural areas, unreliable supply of antihypertensive medications, poor coverage of national health insurance schemes, and underinvestment in health service infrastructure ^16, 19, 21– 23^.

Growing evidence points to the benefits of multifaceted, community-centred interventions involving community health volunteers (CHW) to control non-communicable diseases (NCDs) like hypertension more effectively in resource-constrained settings ^24, 25^. Such interventions were found to be especially promising in rural and remote settings, where access to health facilities is poor ^26– 31^. However, there remain questions about the sustainability and scalability of such programmes, especially where disease burdens are high, but the capacity to effectively address NCDs is low. This requires a deep understanding of the wider context within which interventions are being implemented. Context includes the existing policies, organisational, and institutional structures. Context also includes how people using (patients and their family carers), delivering (health care workers) and overseeing services and structures (decision makers at the different system tiers) understand and experience existing approaches to NCD management, and what changes that introducing a novel intervention will mean for them. Such understanding will be necessary to ensure the acceptability, feasibility, sustainability, and scalability of new service models.

This study is part of a larger project, the National Institute of Health Research (NIHR) Group for Improving Hypertension Control in Rural Sub-Saharan Africa (ICHoR-Africa) ^32^ that seeks to develop and evaluate the feasibility of a community-centred approach to improve the management of people with hypertension in rural Africa. This study will systematically assess the micro (patients/carers), meso (health care workers and facilities) and macro (broader systems) contexts in rural Gambia and Kenya by exploring how people living with hypertension understand and manage their conditions and the perspectives and experiences of those organising, financing, and delivering hypertension care services. It will provide important insights into the perceived and experienced barriers to seeking (people with hypertension) and delivering (health care workers, decision-makers) effective hypertension management and how these can be addressed to inform the development and implementation of a community-centred hypertension management intervention in rural SSA.

### Study aims and objectives

The overarching aim of this study is to understand the experiences, needs, and practices at the individual, organisational, and system levels to detect, treat, and control hypertension in rural Gambia and Kenya. The specific objectives are to:

1. Map a typical journey that someone with hypertension will undertake in each setting and contrast what should happen at the different stages of the journey and what happens in practice.
2. Explore the experiences of people involved in using (patients and their family carers), delivering (health care workers) and overseeing services and structures (decision makers at the different system tiers) for hypertension management.
3. Assess the health services delivery context, including policy and guidelines within which hypertension care is provided.
4. Examine the health system’s capacity and readiness for strengthening the management of hypertension at the different levels of the health system.

## Methods

This is a qualitative study that will use (i) focus group discussion with people living with hypertension and (ii) in-depth interviews with people living with hypertension and their family carers, community health volunteers, health care professionals, decision-makers, and NCD partners in The Gambia and Kenya. We will also review policy documents, guidelines, and standard operating procedures related to hypertension management in both countries. The study period is from August 2022 to April 2024.

### Patient and Public Involvement

Community members and stakeholders have been involved in different stages of developing the IHCoR-Africa study project and its implementation. In the Gambia, this included community health volunteers, managers of health facilities, and representatives of the regional health directorates and the Ministry of Health. In Kenya, this included representatives of community advisory groups, community health volunteers, facility managers, county health managers, Ministry of Health representatives, and NCD partners. We held consultative meetings to discuss the research ideas with members of the community advisory board in Kilifi (Kenya) prior to submitting the study proposal for funding. When funding was granted, planned studies were discussed with various stakeholders at different levels in both countries and their feedback was incorporated into the study protocols before seeking scientific and ethical approvals. Upon securing the necessary approvals implementation of the study activities involved community members and sub-national and national health authorities. In both countries, community members are actively involved in sensitisation and consenting of study participants. The findings of this study will also be discussed with community members and stakeholders for validation during meetings. Furthermore, dissemination workshops will be organised at different levels in both countries.

### Conceptual framing

Our overall approach to this study places people with hypertension (and their family carers) at the centre of the hypertension care pathway. It recognises that availability of, and access to services that are necessary for successful hypertension management while noting that the social and economic contexts within which people live greatly impact their capacity to seek care and self-manage their conditions. This creates a ‘treatment burden’ interacting with and complicating clinical care ^33^. High burdens will likely result in poorer health care outcomes for individuals. Our analytical approach will be guided by the Cumulative Complexity Model (CCM) ^34^. The CCM distinguishes the demands placed upon people with chronic health problems to (self-) manage their conditions alongside other daily tasks that are associated with living with a chronic disease. This involves users having to engage with health services (‘patient workload’) and the capacity of health facilities to meet patients’ needs given their physical and mental well-being, levels of literacy and beliefs, and importantly, their wider social and economic environment and resources available to them (‘patient capacity’). Patient workload and capacity are dynamic and interact; an imbalance between these will create compounded risks in care-seeking (e.g., low adherence to treatment) and outcomes (such as complications). Emerging evidence drawing on this model in SSA found how clinical and social factors accumulate and interact to complicate care for people with chronic conditions ^35– 38^.

We will also draw on components of implementation research, in particular theories of organisational readiness and the dissemination of innovations ^39, 40^. This will enable us to understand the motivations for and capacity to implement the changes required at the organisational and system levels to improve hypertension management in rural Kenya and Gambia.

### Settings

This study will be conducted in the Lower River Region of the Gambia (West Africa) and in Kilifi County in Kenya (East Africa). Both settings have high levels of hypertension, estimated to range from 18.3%–32.4% in The Gambia ^41^ and 12.6–36.9% in Kenya ^42^. Documented prevalence rates are likely to be underestimated with data from The Gambia suggesting that almost 70% of those with hypertension may be undiagnosed ^43^. Treatment and control rates are low; for example, in The Gambia, in 2018, an estimated 32.5% of hypertensive patients were on treatment and only 10% had their BP controlled ^44^. In Kilifi County, only 3% of those diagnosed have their BP controlled ^45^.

Both settings have a well-developed research infrastructure which is critical for the overall project activities: Medical Research Council (MRC) Unit The Gambia at LSHTM in Kiang West, and KEMRI Wellcome Trust Research Programme (KWTRP) in Kilifi.

***The Gambia.*** The Gambian operates a three-tier system for the delivery of public health services. The primary level (village health services) is the first point of contact and is run by two community health volunteers, village health workers (VHW) and community birth companions (CBC) ^46^. Some communities also have a community clinic run by community health nurses (CHN) or a minor health centre, providing a wider range of services to people who cannot be managed by the VHWs/CBCs ^46^. The secondary level comprises (i) major health centres that receive patients on referral from community clinics and minor health centres ^46^ and (ii) district hospitals which are referral points for minor and major health centres; they offer comprehensive emergency obstetric and surgery services including caesarean section and blood transfusion. The tertiary level consists of thegeneral, teaching and, specialised hospitals; teaching and specialised hospitals are the highest level of the referral system. District hospitals are supervised by the Regional Health Directorate, whereas general and teaching hospitals are semi-autonomous ^46^.

The population of the Lower River Region was estimated at 89,157 in 2020 ^47^. It is mainly rural, with high levels of poverty, at around 60% in 2020 ^48^. Health services are provided by 13 public health facilities, including 7 community clinics, 5 minor health centres and one district hospital. Facility staffing (skilled health workers) is at 1.54/1000 population, slightly higher than the national average at 1.53/1000 population ^47^ but significantly lower than the WHO recommended standard of 4.45/1000 ^49^.

***Kenya.*** The delivery of public health services in Kenya is organised in six levels: community health services (level 1), primary care services provided by dispensaries (level 2) and health centres (level 3), primary referral hospitals (level 4), secondary referral hospitals (level 5) and tertiary (national) referral hospitals (level 6) that provide highly specialised services including specialist medical care, laboratory support, blood product services, and research ^50, 51^. In Kenya, health facility staffing is at 3.01/1,000 individuals ^52^, which is lower than the WHO recommendation of 4.45/1,000 ^49^.

Kilifi County has a population of about 1.4 million (2019) ^53^. Mainly rural, with main economic activities being subsistence farming, fishing, and tourism ^54^, Kilifi is among the poorest counties in Kenya, with around half of the population unable to afford the basic basket of goods in 2021 ^55^. Health care services in Kilifi County are provided by 150 public health facilities, including 132 dispensaries, 9 health centres, and 9 primary referral hospitals ^56^. The County has a total of 1,522 health care workers as of December 2022, with 5.96 skilled health workers per 10,000 people ^52, 57^.

### Data collection methods

The following data collection methods will be used: in-depth interviews with patients, health care workers, decision-makers and NCD partners, focus group discussions with patients, and document review. Different data types will contribute to different objectives.

***Focus group discussions (FGD) (Objective 1).*** Focus group discussions (FGD) aim to understand how people living with hypertension interact with health care services, from the point of symptom recognition, screening, and diagnosis to ongoing treatment. This first step will allow us to generate a mapping of the hypertension patient journey, assuming a ‘typical patient’, but noting important variations or divergences as well as gaps in the journey where necessary. It will inform work for Objectives 2–4, and a detailed explanatory analysis, which will be important in understanding points of variation in the journey, major overlaps and gaps as well as giving insights into how the journey is experienced.

***In-depth interviews (IDI) (Objectives 2–4).*** We will use in-depth interviews to enable a detailed understanding of the views and experiences of those living with hypertension (Objective 2) and of those providing and/or overseeing care for people with hypertension (Objective 3) as well as exploration of the readiness for and capacity of health facilities to introduce new ways of working to improve hypertension management in the two study settings (Objective 4). In-depth interviews provide a suitable approach to engage with people’s accounts of their experiences and help understand the context (home, family, work) in which study participants deal with issues related to their health condition. This approach will generate data on how patients pass through the health care system and the challenges they encounter along the care pathway, from the service user, provider, and decision-maker perspectives, for managing their hypertension, access to and use of services, treatment adherence, and patients’ and health care workers’ preferences for care.

***Document review (Objectives 1 and 3).*** The document review aims to map service delivery structures, policies and frameworks and the journey of patients with hypertension through health care system in both countries. We will review (i) the principal organisation of the service delivery structures in Kilifi (Kenya) and Lower River Region (The Gambia), including current strategies for and approaches to referral generally and (ii) national/local level guidance on hypertension screening, diagnosis, and management specifically. We will analyse published and unpublished documents including national-level strategy and policy documents, hypertension guidelines issued by professional associations, local standard operating procedures, and other relevant documentation in each study site. Documents will be identified through targeted searches of the websites of governmental and non-governmental agencies and organisations with a remit in health service and system organisation, financing, and governance, and NCDs specifically, including Departments of Ministry of Health in both countries, The Gambia Alcohol Policy Alliance, Pan African Society of Cardiology, the Kenya Cardiac Society, and the NCD Alliance of Kenya. These organisations are project partners; we will consult with them directly to identify any additional information that is not in the public domain. We will also conduct Google Scholar searches to identify any additional documents of relevance to the study aim using the following search terms “guideline/s or protocol”, “hypertension”, “diagnosis”, “treatment”, “control” and “sub-Sahara/n Africa”. Furthermore, we will approach individual health facilities in the study locations in Kenya and The Gambia to collate clinical protocols and standard operating procedures relating to the management of hypertension where these are available. For the purposes of this study, we understand ‘management’ to encompass the diagnosis and treatment of hypertension We will use a template to extract data from the included documents for the analysis.

### Data collection

***Recruitment of FGD and interview participants.*** We will use a combination of purposive and snowball sampling to identify FGD (people with hypertension and their family carers) and interview participants (people with hypertension/carers, health care workers involved in the delivery of hypertension care, community health volunteers, and decision-makers).

***Patients.*** We will recruit a range of patients from different socio-demographic backgrounds, their location in the patient journey (newly diagnosed, commenced treatment, with complications, drop out, etc.), as well as geographic location (distance to health facilities). For both focus group discussions and in-depth interviews, patients will be identified through the following ways.

i) we will use an opportunistic sampling approach by visiting health facilities on hypertension clinic days (Kenya only; health facilities in The Gambia study site do not operate disease-specific clinic days). People with hypertension visiting the facility on that day will be contacted for participation in the study.
ii) We will work with village health workers (VHW) in The Gambia or community health promoters (CHP) in Kenya to identify and approach patients with hypertension. VHW/CHP are appointed by their local community members and work with them as health volunteers.
iii) We will snowball from patients who have agreed to participate in our data collection.

***Health care workers.*** We will recruit a purposive sample of health care workers involved in the diagnosis, treatment, and control of hypertension across the different levels of service delivery, including physicians, clinical officers, nurses, and community health volunteers in both study settings.

In The Gambia (Lower River Region), we will invite health care workers involved in hypertension management at village health services, community clinics and minor health centres (level 1), and the district hospital (level 2). In Kenya (Kilifi), we will consider all levels of health service delivery, including community health services (level 1), dispensaries (level 2), health centres (level 3), as well as sub-county and county hospitals (levels 4 and 5).

In both study sites, the selection of facilities to be included in the study will be discussed with the relevant health authorities (Gambia Lower River Regional health authorities and Kilifi County health authorities in Kenya) to identify those with active hypertension management clinics. At each level of health facility, the number of health care workers to include in the data collection will be 1-3 individuals.

***Decision-makers.*** These will include those representing the different governance levels in each study site, that is, senior executives (stakeholders involved in NCD/hypertension policy including policy formulation, adoption, implementation, and oversight at national and sub-national levels), managers of health facilities, and members of professional organisations involved in hypertension care programmes. We anticipate that a small number of health care workers identified above will also be facility managers; interviewing them will aim to explore their dual role as both service providers and decision-makers.

The sampling frame for this study is purposive rather than representative. It is intended to capture the range of perspectives of those directly or indirectly involved in hypertension diagnosis and management. Table 1 indicates the sampling for this study.

**Table 1.** Sampling for this study.

Country	Data collection approach	Type of participant	Sample
Gambia	In-depth interview	Patients	25–30
		Health care workers	15–20
		Village Health Workers	5–10
		Decision makers	10–15
	Focus group discussion	Patients	20–32
Kenya	In-depth interview	Patients	25–30
		Health care workers	15–20
		Community Health Promoters	5–10
		Decision makers	10–15
	Focus group discussion	Patients	20–32
**Total (both countries)**			**150–214**

In total, eight focus group discussions (FGDs) with patients assisted by their adult family carers (where feasible) will be conducted. The overall number of participants for the eight FGDs across the two countries will be 40 – 64 individuals, that is, 20 – 32 participants in each study site.

Regarding in-depth interviews, we anticipate including 110 – 150 participants in total across the two countries, that is, 55 – 75 participants in each.

Qualitative sample sizes are best determined iteratively when themes generated in interviews approach saturation, based on the judgement and experience of interviewers ^58^. Data saturation will be assessed on the basis of whether the study team is not able to identify any new themes after conducting a certain number of consecutive interviews in each category of informants in each study setting. This means that recruitment will stop when data that has been collected is viewed as adequate in relation to the study objectives and that any further data collection will not generate any additional insights. Therefore, for all participant categories, the final number of informants may vary, which means, we might include a higher or lower number of participants in each category as data collection progresses. This means that additional participants may be identified during data collection who will then be approached for interviews.

### FGD and interview process

We developed five topic guides for the different stakeholder groups and interview forms (FGD, IDI) to gain insight into the lived experiences of patients, health care workers, and decision-makers with hypertension care with regard to service use, provision, and oversight, drawing on phenomenological enquiry.

The development of topic guides for patients was informed by the Cumulative Complexity Model ^34^ to further explore the experienced burdens of people with chronic diseases such as hypertension in managing their conditions alongside their daily tasks in their communities. The tools further explore people’s journey through the system from the first time they were diagnosed, follow-up visits, treatment, and medication, and management of any complications related to their condition to the involvement of community health volunteers in the diagnosis and management of hypertension in their communities.

Topic guides for interviews with village health workers/community health promoters, health care workers and decision-makers seek to understand their views on the services for hypertension management, including existing treatment protocols and guidelines; experiences of the organisation and delivery of health services for the diagnosis, treatment, and control of hypertension, as well as perceptions of barriers and opportunities to improve current approaches to hypertension care in rural Gambia and Kenya. We also explore perceptions of the capacity and readiness of health systems to improve the delivery of hypertension care. Topic guide development drew on theories of organisational readiness and the dissemination of innovations ^39, 40^, enabling us to understand how services in both countries may be able to accommodate or implement the changes required at the organisational and system level to improve hypertension management in rural Kenya and Gambia.

An FGD session will convene 5–8 people with hypertension and family carers (depending on the willingness of the patient to be assisted) and will last between 60 to 90 minutes. Four FGDs will be organised in both sites, two FGDs with females and two others with males, in each site.

In-depth interviews will last between 30 to 60 minutes and be carried out face-to-face.

An experienced team in qualitative research will collect data from participants in local languages (Swahili or Giriama in Kenya and Mandinka or Wolof in The Gambia) or in English depending on the participant’s language preference. Both in-depth interviews and focus group discussions will take place in a location most convenient for participants. During fieldwork, we will hold review meetings to reflect on our findings and experiences and adjust data collection strategies as needed.

### Data analysis

Interviews and focus group discussions will be recorded, with participants’ permission, and transcribed verbatim in English. Transcripts will be imported into the software NVivo 12 for processing and the data will be analysed thematically. Transcripts will be read repeatedly to familiarise ourselves with the data; data will be inductively coded to develop the coding frame. The resulting coding frame will be applied iteratively across the transcripts and modifications will be made depending on the data, as themes reoccur and appear important about the lived experiences of study participants. These codes will then be grouped into overarching themes, which will be analysed to compare findings across cases. This stage of analysis will involve discussions within the research team to refine the themes and to group them into meaningful conceptual categories. This will allow us to draw tentative conclusions about aspects of the lived experiences of patients with hypertension and their care-seeking from health facilities, experiences and expectations of health care workers and decision-makers about better organisation and delivery of hypertension care in rural settings. The data analysis will further be guided by the Cumulative Complexity Model ^34^, organisational readiness, and the dissemination of innovations theories ^39, 40^ as indicated above.

### Ethical approval

Approvals have been granted by the MRCG Scientific Coordination Committee and The Gambia Government – MRCG joint Ethics Committee in The Gambia (ref. 28313, January 4 ^th^, 2023); the KEMRI Scientific Committee and the Scientific and Ethics Review Unit (SERU) in Kenya (ref. 4631, February 27 ^th^ 2023); the LSHTM Ethics Committee in the UK (ref. 28313, 7 February 7 ^th^, 2023).

Informed consent will be obtained from all participants in this study through their signatures or fingerprints on an informed consent form, in which participants confirm that they have read and understood the project information sheet. Additionally, at the beginning of each interview or FGD, the interviewer/FGD moderator will seek confirmation from participants that they are willing to participate in the data collection and that they agree that the interview and FGD will be audio recorded and transcribed. In case participants are unable to read the information sheet and consent form, these will be read out to them in their native language in the presence of a witness and their consent will be sought and obtained through their signatures or fingerprints on the forms; where a suitable witness cannot be identified, the participant will not be interviewed. After the consenting, a copy of the forms will be given to the participant to keep, and the study team will keep another copy for their record.

Data collected in each study country will be kept in a secure password-protected environment and any hard copies of interview transcripts and consent forms will be kept in a locked cabinet. Once data have been processed and databases created, they will be encrypted, password-protected and saved on a secured server under each institutional data protection policies, i.e. MRC Gambia (MRCG) and KEMRI-Wellcome Trust Research Programme (KWTRP). Encrypted data from both study sites excluding any personal identifiers will later be transferred to a secure server at LSHTM to be used by researchers across different sites for cross-country analysis.

### Dissemination

The findings of this study will inform the development of a community-based intervention in a separate study protocol using a participatory action research approach ^59^ to be implemented for the diagnosis, treatment, and control of hypertension in rural Gambia and Kenya, as part of the broader IHCoR-Africa research project. Findings and learning will be shared with health care workers, community members, administrative and political stakeholders involved in decision-making about hypertension management policies and guidelines, and members of professional organisations involved in hypertension care programmes through workshops at national and subnational levels. The findings will also be made available on the IHCoR-Africa website. Furthermore, findings will be disseminated via peer-reviewed publications, conferences, media, and lay reports.

## Discussion

This study is conducted as part of the broad IHCoR-Africa study that seeks to develop, implement, and evaluate a community-centred intervention to improve hypertension management in rural Gambia and Kenya. Drawing on the phenomenological enquiry approach, this study will provide a genuine understanding of different aspects of hypertensive patients' encounters along the care pathway and the system-wide contextual factors from the perspective of hypertension care users, providers, and decision-makers. It will also shed light on the existing organisational structure for diagnosis, treatment, and control of hypertension and how these can be improved to meet the health care needs of hypertensive patients in rural settings. This will significantly contribute to the understanding of barriers to and facilitators of effective hypertension management at individual, health service, and system levels, essential for developing an innovative approach to improve hypertension diagnosis, treatment, and control. Another strength of this study is the analytical framework that it draws on, that is, the organisational readiness and dissemination of innovations theories. This will provide evidence on the capacity and readiness of health systems in both countries to strengthen the management of hypertension at different levels of care delivery and highlight how implementing a community-centred intervention interacts with the broader system. This is essential for ensuring the acceptability, feasibility, sustainability, and scalability of new service models to be delivered and embedded into existing systems for effective hypertension diagnosis, treatment, and control in rural settings.

### Study status

Ethics approvals have been secured in both countries and participants' recruitment to the study began in July 2023 as well as data collection. In Kenya, the data collection is ongoing but was completed in The Gambia in October 2023. The transcription of audio files is ongoing in both countries, after which the data analysis will take place.

## Conclusions

Using a pragmatic and comprehensive methodological approach, this study will provide insights into the ways health systems in the two countries are ready for change to improve hypertension management. It will also inform the development and implemention of a community-centred intervention and its integration into existing systems in ways that ensure its sustainability and scalability in rural settings.

## Data Availability

No data are associated with this article.

## References

[1] Mills KT Stefanescu A He J : The global epidemiology of hypertension. *Nat Rev Nephrol.* 2020;16(4):223–237. 10.1038/s41581-019-0244-2 32024986 PMC7998524

[2] Zhou B Bentham J Di Cesare M : Worldwide trends in blood pressure from 1975 to 2015: a pooled analysis of 1479 population-based measurement studies with 19·1 million participants. *Lancet.* 2017;389(10064):37–55. 10.1016/S0140-6736(16)31919-5 27863813 PMC5220163

[3] Metrics IfH, Evaluation: Global Burden of Disease: GBD compare data Visualization. Seattle, WA: IHME, University of Washington,2022. Reference Source

[4] Adeloye D Basquill C : Estimating the prevalence and awareness rates of hypertension in Africa: a systematic analysis. *PLos One.* 2014;9(8): e104300. 10.1371/journal.pone.0104300 25090232 PMC4121276

[5] Ataklte F Erqou S Kaptoge S : Burden of undiagnosed hypertension in sub-saharan Africa: a systematic review and meta-analysis. *Hypertension.* 2015;65(2):291–8. 10.1161/HYPERTENSIONAHA.114.04394 25385758

[6] Cappuccio FP Miller MA : Cardiovascular disease and hypertension in Sub-Saharan Africa: burden, risk and interventions. *Intern Emerg Med.* 2016;11(3):299–305. 10.1007/s11739-016-1423-9 27001886 PMC4820479

[7] Gnugesser E Chwila C Brenner S : The economic burden of treating uncomplicated hypertension in Sub-Saharan Africa: a systematic literature review. *BMC Public Health.* 2022;22(1): 1507. 10.1186/s12889-022-13877-4 35941626 PMC9358363

[8] Yusuf S Rangarajan S Teo K : Cardiovascular risk and events in 17 low-, middle-, and high-income countries. *N Engl J Med.* 2014;371(9):818–27. 10.1056/NEJMoa1311890 25162888

[9] Wozniak G Khan T Gillespie C : Hypertension control cascade: a framework to improve hypertension awareness, treatment, and control. *J Clin Hypertens (Greenwich).* 2016;18(3):232–9. 10.1111/jch.12654 26337797 PMC5049660

[10] Herbst AG Olds P Nuwagaba G : Patient experiences and perspectives on hypertension at a major referral hospital in rural southwestern Uganda: a qualitative analysis. *BMJ Open.* 2021;11(1): e040650. 10.1136/bmjopen-2020-040650 33408202 PMC7789452

[11] Lasco G Mendoza J Renedo A : *Nasa dugo* ('It's in the blood'): lay conceptions of hypertension in the Philippines. *BMJ Global Health.* 2020;5(7): e002295. 10.1136/bmjgh-2020-002295 32646854 PMC7351273

[12] Lynch HM Green AS Nanyonga RC : Exploring patient experiences with and attitudes towards hypertension at a private hospital in Uganda: a qualitative study. *Int J Equity Health.* 2019;18(1): 206. 10.1186/s12939-019-1109-9 31888767 PMC6937689

[13] Manavalan P Minja L Wanda L : "It's because I think too much": perspectives and experiences of adults with hypertension engaged in HIV care in northern Tanzania. *PLoS One.* 2020;15(12): e0243059. 10.1371/journal.pone.0243059 33270765 PMC7714125

[14] Naanyu V Vedanthan R Kamano JH : Barriers influencing linkage to hypertension care in Kenya: qualitative analysis from the LARK hypertension study. *J Gen Intern Med.* 2016;31(3):304–14. 10.1007/s11606-015-3566-1 26728782 PMC4762819

[15] Bay N Juga E Macuacua C : Assessment of care provision for hypertension at the emergency Department of an Urban Hospital in Mozambique. *BMC Health Serv Res.* 2019;19(1): 975. 10.1186/s12913-019-4820-8 31852481 PMC6921411

[16] Heller DJ Kumar A Kishore SP : Assessment of barriers and facilitators to the delivery of care for noncommunicable diseases by nonphysician health workers in low- and middle-income countries: a systematic review and qualitative analysis. *JAMA Netw Open.* 2019;2(12): e1916545. 10.1001/jamanetworkopen.2019.16545 31790570 PMC6902752

[17] Khatib R Schwalm JD Yusuf S : Patient and healthcare provider barriers to hypertension awareness, treatment and follow up: a systematic review and meta-analysis of qualitative and quantitative studies. *PLoS One.* 2014;9(1): e84238. 10.1371/journal.pone.0084238 24454721 PMC3893097

[18] Mulugeta TK Kassa DH : Readiness of the primary health care units and associated factors for the management of hypertension and type II diabetes mellitus in Sidama, Ethiopia. *PeerJ.* 2022;10: e13797. 10.7717/peerj.13797 36042860 PMC9420406

[19] Musinguzi G Bastiaens H Wanyenze RK : Capacity of health facilities to manage hypertension in Mukono and Buikwe districts in Uganda: challenges and recommendations. *PLoS One.* 2015;10(11): e0142312. 10.1371/journal.pone.0142312 26560131 PMC4641641

[20] Peck R Mghamba J Vanobberghen F : Preparedness of Tanzanian health facilities for outpatient primary care of hypertension and diabetes: a cross-sectional survey. *Lancet Glob Health.* 2014;2(5):e285–e92. 10.1016/S2214-109X(14)70033-6 24818084 PMC4013553

[21] Barasa E Kazungu J Nguhiu P : Examining the level and inequality in health insurance coverage in 36 Sub-Saharan African countries. *BMJ Global Health.* 2021;6(4): e004712. 10.1136/bmjgh-2020-004712 33903176 PMC8076950

[22] Husain MJ Datta BK Kostova D : Access to cardiovascular disease and hypertension medicines in developing countries: an analysis of essential medicine lists, price, availability, and affordability. *J Am Heart Assoc.* 2020;9(9): e015302. 10.1161/JAHA.119.015302 32338557 PMC7428558

[23] Risso-Gill I Balabanova D Majid F : Understanding the modifiable health systems barriers to hypertension management in Malaysia: a multi-method health systems appraisal approach. *BMC Health Serv Res.* 2015;15: 254. 10.1186/s12913-015-0916-y 26135302 PMC4489127

[24] Khan M Lamelas P Musa H : Development, testing, and implementation of a training curriculum for nonphysician health workers to reduce cardiovascular disease. *Glob Heart.* 2018;13(2):93–100. e1. 10.1016/j.gheart.2017.11.002 29331282 PMC6090270

[25] Schwalm JDR McCready T Lamelas P : Rationale and design of a cluster randomized trial of a multifaceted intervention in people with hypertension: the Heart Outcomes Prevention and Evaluation 4 (HOPE-4) Study. *Am Heart J.* 2018;203:57–66. 10.1016/j.ahj.2018.06.004 30015069

[26] Jafar TH Gandhi M De Silva HA : A community-based intervention for managing hypertension in rural South Asia. *N Engl J Med.* 2020;382(8):717–26. 10.1056/NEJMoa1911965 32074419

[27] Jafar TH Hatcher J Poulter N : Community-based interventions to promote blood pressure control in a developing country: a cluster randomized trial. *Ann Intern Med.* 2009;151(9):593–601. 10.7326/0003-4819-151-9-200911030-00004 19884620

[28] Joshi R Alim M Kengne AP : Task shifting for non-communicable disease management in low and middle income countries--a systematic review. *PLoS One.* 2014;9(8): e103754. 10.1371/journal.pone.0103754 25121789 PMC4133198

[29] Schwalm JD McCready T Lopez-Jaramillo P : A community-based comprehensive intervention to reduce cardiovascular risk in hypertension (HOPE 4): a cluster-randomised controlled trial. *Lancet.* 2019;394(10205):1231–42. 10.1016/S0140-6736(19)31949-X 31488369

[30] Some D Edwards JK Reid T : Task shifting the management of non-communicable diseases to nurses in Kibera, Kenya: *does it work?* *PLoS One.* 2016;11(1): e0145634. 10.1371/journal.pone.0145634 26812079 PMC4727908

[31] Vedanthan R Kumar A Kamano JH : Effect of Nurse-Based management of hypertension in rural Western Kenya. *Glob Heart.* 2020;15(1):77. 10.5334/gh.856 33299773 PMC7716784

[32] IHCoR-Africa: A NIHR global research group on improving hypertension control in rural Sub-Saharan Africa.2023. Reference Source

[33] May CR Eton DT Boehmer K : Rethinking the patient: using burden of treatment theory to understand the changing dynamics of illness. *BMC Health Serv Res.* 2014;14: 281. 10.1186/1472-6963-14-281 24969758 PMC4080515

[34] Shippee ND Shah ND May CR : Cumulative complexity: a functional, patient-centered model of patient complexity can improve research and practice. *J Clin Epidemiol.* 2012;65(10):1041–51. 10.1016/j.jclinepi.2012.05.005 22910536

[35] Angwenyi V Aantjes C Kajumi M : Patients experiences of self-management and strategies for dealing with chronic conditions in rural Malawi. *PLoS One.* 2018;13(7): e0199977. 10.1371/journal.pone.0199977 29965990 PMC6028088

[36] Chikumbu EF Bunn C Kasenda S : Experiences of multimorbidity in urban and rural Malawi: an interview study of burdens of treatment and lack of treatment. *PLOS Global Public Health.* 2022;2(3): e0000139. 10.1371/journal.pgph.0000139 36962280 PMC10021162

[37] Matima R Murphy K Levitt NS : A qualitative study on the experiences and perspectives of public sector patients in Cape Town in managing the workload of demands of HIV and type 2 diabetes multimorbidity. *PLoS One.* 2018;13(3): e0194191. 10.1371/journal.pone.0194191 29538415 PMC5851623

[38] Van Pinxteren M Mbokazi N Murphy K : The impact of persistent precarity on patients’ capacity to manage their treatment burden: a comparative qualitative study between urban and rural patients with multimorbidity in South Africa. *Front Med (Lausanne).* 2023;10: 1061190. 10.3389/fmed.2023.1061190 37064034 PMC10098191

[39] Falchetta G Hammad AT Shayegh S : Planning universal accessibility to public health care in sub-Saharan Africa. *Proc Natl Acad Sci U S A.* 2020;117(50):31760–31769. 10.1073/pnas.2009172117 33257557 PMC7749323

[40] Greenhalgh T Robert G Macfarlane F : Diffusion of innovations in service organizations: systematic review and recommendations. *Milbank Q.* 2004;82(4):581–629. 10.1111/j.0887-378X.2004.00325.x 15595944 PMC2690184

[41] Koller R Agyemang C : Prevalence of cardiovascular disease risk factors in the Gambia: a systematic review. *Glob Heart.* 2020;15(1): 42. 10.5334/gh.827 32923336 PMC7427677

[42] Mogaka JN Sharma M Temu T : Prevalence and factors associated with hypertension among adults with and without HIV in Western Kenya. *PLos One.* 2022;17(1): e0262400. 10.1371/journal.pone.0262400 35007291 PMC8746744

[43] Cham B Scholes S Ng Fat L : Burden of hypertension in The Gambia: evidence from a national World Health Organization (WHO) STEP survey. *Int J Epidemiol.* 2018;47(3):860–871. 10.1093/ije/dyx279 29394353

[44] Modou Jobe ea: Gambian national eye health survey (2018).2022.

[45] Etyang AO Munge K Bunyasi EW : Burden of disease in adults admitted to hospital in a rural region of coastal Kenya: an analysis of data from linked clinical and demographic surveillance systems. *Lancet Glob Health.* 2014;2(4):e216–e24. 10.1016/S2214-109X(14)70023-3 24782954 PMC3986034

[46] Ministry of Health: National multi-sectoral strategy and costed action plan for non-communicable disease prevention and control in The Gambia 2022-2027.. In: Non-Communicable Disease Programme Unit, Directorate of Health Promotion and Education, editor. Ministry of Health, Banjul: Republic of The Gambia;2022. Reference Source

[47] Health Mo: 2020 Service statistics report. Health Management Information System (HMIS). Banjul, The Gambia: Ministry of Health, Information UDoPa;2021. Reference Source

[48] Touray S : Poverty trends in The Gambia: a tale of two crises. World Bank, June 26,2023. Reference Source

[49] World Health Organization: Global strategy on human resources for health: workforce 2030.2016. Reference Source

[50] Republic K: Kenya health policy 2012-2030. In: Sanitation MoMSMoPHa, editor. Nairobi,2012.

[51] Republic K: Kenya universal health coverage policy 2020-2030. accelerating attainment of universal health coverage. In: Health Mo editor. Nairobi: Ministry of Health;2020. Reference Source

[52] Okoroafor SC Kwesiga B Ogato J : Investing in the health workforce in Kenya: trends in size, composition and distribution from a descriptive health labour market analysis. *BMJ Glob Health.* 2022;7(Suppl 1): e009748. 10.1136/bmjgh-2022-009748 36008084 PMC9422806

[53] KNBS: Kenya population and housing census. Nairobi, Kenya.;2019. Reference Source

[54] Scott JAG Bauni E Moisi JC : Profile: the Kilifi Health and Demographic Surveillance System (KHDSS). *Int J Epidemiol.* 2012;41(3):650–7. 10.1093/ije/dys062 22544844 PMC3396317

[55] KNBS: The Kenya poverty report. Based on the 2021 Kenya continuous household survey. In: Statistics KNBo, editor. Nairobi: Kenya National Bureau of Statistics, World Bank Group;2021. Reference Source

[56] Tsofa B Omondi L Ngala T : Kilifi county health sector review. In: Government KC, editor. Kilifi: Kilifi County Government;2023.

[57] CGoK: Kilifi county integrated development plan III 2023-2027. Accelerating socioeconomic transformation for inclusive growth. In: Kilifi CGo, editor. Kilifi,2023. Reference Source

[58] Bakibinga P Kisia L Atela M : Demand and supply-side barriers and opportunities to enhance access to healthcare for urban poor populations in Kenya: a qualitative study. *BMJ Open.* 2022;12(5): e057484. 10.1136/bmjopen-2021-057484 35523490 PMC9083429

[59] Cornish F Breton N Moreno-Tabarez U : Participatory action research. *Nat Rev Methods Primers.* 2023;3(1):34. 10.1038/s43586-023-00214-1

