# Supplementary material for: Managing hypertension in rural Gambia and Kenya: Protocol for a qualitative study exploring the experiences of patients, health care workers, and decision-makers
Source: NIHR Open Res. 2024 Aug 19;4:5. Originally published 2024 Feb 16. [Version 3] doi: 10.3310/nihropenres.13523.3 (PMC11375402; doi:10.3310/nihropenres.13523.3)
Supplement: Peer review history

# Reviewer report: Reviewer response for version 3

**DOI:** 10.3310/nihropenres.14902.r32585

**Published:** 2024-09-04

**Authors:** Hon Lon Tam

**Affiliations:** 1 The Chinese University of Hong Kong, Hong Kong, Hong Kong

My concerns were well addressed. I am fine with the revision.

Is the study design appropriate for the research question?

Yes

Is the rationale for, and objectives of, the study clearly described?

Yes

Are sufficient details of the methods provided to allow replication by others?

Partly

Are the datasets clearly presented in a useable and accessible format?

Yes

Reviewer Expertise:

cardiometabolic disease  management

I confirm that I have read this submission and believe that I have an appropriate level of expertise to confirm that it is of an acceptable scientific standard.

# Reviewer report: Reviewer response for version 2

**DOI:** 10.3310/nihropenres.14758.r32420

**Published:** 2024-08-06

**Authors:** Hon Lon Tam

**Affiliations:** 1 The Chinese University of Hong Kong, Hong Kong, Hong Kong

Hypertension is a global health issue. This study will provide a comprehensive understanding regarding the hypertension management in Gambia and Kenya. But I have some comments for the authors to consider.

 1. Gambia and Kenya are located in different regions in Africa. They may have different cultures that affect the hypertension management. Please describe if these countries are suitable to analyze together.

 2. Please describe what "phenomenological enquiry" will be used?

Is the study design appropriate for the research question?

Yes

Is the rationale for, and objectives of, the study clearly described?

Yes

Are sufficient details of the methods provided to allow replication by others?

Partly

Are the datasets clearly presented in a useable and accessible format?

Yes

Reviewer Expertise:

cardiometabolic disease  management

I confirm that I have read this submission and believe that I have an appropriate level of expertise to confirm that it is of an acceptable scientific standard, however I have significant reservations, as outlined above.

# Reviewer report: Reviewer response for version 2

**DOI:** 10.3310/nihropenres.14758.r31545

**Published:** 2024-04-25

**Authors:** Olutobi Sanuade

**Affiliations:** 1 Spencer Fox Eccles School of Medicine at the University of Utah,, Salt Lake, USA

I have gone through the revision, and authors have sufficiently addressed all the comments. So, I have no further comments to make.

Is the study design appropriate for the research question?

Yes

Is the rationale for, and objectives of, the study clearly described?

Yes

Are sufficient details of the methods provided to allow replication by others?

Partly

Are the datasets clearly presented in a useable and accessible format?

Not applicable

Reviewer Expertise:

Cardiovascular health (hypertension), implementation science , qualitative research, mixed-methods, health disparities.

I confirm that I have read this submission and believe that I have an appropriate level of expertise to confirm that it is of an acceptable scientific standard.

# Reviewer report: Reviewer response for version 1

**DOI:** 10.3310/nihropenres.14677.r31190

**Published:** 2024-03-07

**Authors:** Olutobi Sanuade

**Affiliations:** 1 Spencer Fox Eccles School of Medicine at the University of Utah,, Salt Lake, USA

**Summary of the article**

 This study aims to systematically assess the micro (patients/carers), meso (health care workers

 and facilities) and macro (broader systems) contexts in rural Gambia and Kenya by exploring how people living with hypertension understand and manage their conditions and the perspectives and experiences of those organizing, financing, and delivering hypertension care services. This study is very relevant given the high prevalence of hypertension in sub-Saharan Africa and the potential impact it can have on the population. Understanding ways to enhance hypertension care in SSA is crucial, and this study will offer valuable insights on how to achieve that.

 While the paper provides valuable insights into the topic at hand, there are areas where improvements can be made to ensure its scientific rigor. Here are some suggestions for enhancing the paper's scientific credibility.

**Abstract**

- ***Background: *** The objective stated here seems to be incomplete. Please ensure that it is the same with the one state in the introduction section.
- ***Methods***: Authors should please provide the methods that they intend to use to assess the capacity and readiness of the health systems.
- ***Expected findings: *** Will this study also be able to describe context for hypertension control since the national guidelines and policy documents will be able to provide this information? Authors need to be consistent as to what the focus of their study will be. Will this study address hypertension diagnosis, management and control, or hypertension diagnosis or diagnosis and management?

**Introduction**

- “ *Hypertension is a major cause of premature mortality……an estimated 10.7 million deaths globally”* Please is this estimate annual estimate?
- “ *Sub-Saharan African (SSA) has among them highest.....” *Please edit this sentence for clarity. I will also suggest breaking this into two sentences to improve clarity.

**Methods**

- *"This is a qualitative study that will use focus group discussion and in-depth interviews with people living with hypertension….” *The participant focus here is different from what was stated in the abstract. Please reconcile both.
- ***Patient and public involvement:*** “ *Community members and stakeholders….” *Please provide the list of these stakeholders.
- ***Conceptual framing*** **:** “Our overall approach to this study…..” It seems this sentence is incomplete. Authors should please state what they mean by their approach placing patients at the centre. At the centre of what? policy focus?
- ***Conceptual framing: …***..” *to improve hypertension management and control in rural Kenya and Gambia” *How about hypertension detection? Authors need to be clear with the focus of their study- Are they interested in improving hypertension diagnosis, management and control, or only management and control? This inconsistency seems to run through the paper.
- ***Data collection methods:*** Since Kenya and the Gambia operate different healthcare system, how did authors decide the level to choose health care worker participants from in these two countries? I think some statements around this will be helpful to readers.
- ***Data collection methods-IDI: **“…as well as exploration of the readiness for and capacity of health facilities….” *Please which level of health facilities are the authors referring TO here, given that there are different levels and these vary in Kenya and Gambia?
- ***Document Review (objectives 1 and 3): **“We will analyse published and unpublished documents including national-level strategy….”* This approach is different from what was stated in the abstract. Please reconcile this.
- ***Document Review (objectives 1 and 3)***: “ *We will also conduct Google scholar searches…” *Please what will the search term focus on?
- ***Document Review (objectives 1 and 3):*** *“…and The Gambia to collate clinical protocols and standard operating procedures as they relate to hypertension control”* In sub-Saharan Africa, standard operating procedures are usually not written down especially in lower-tier facilities. In such situation, how will the authors obtain the clinical protocols and standard operating procedures?
- ***Data collection/health care workers: *** “ *In the Gambia (Lower River Region), we will invite health care workers involved in hypertension….” *Authors should please provide the number of participants that they intend to recruit from each of these levels of care.
- ***FGD and interview process:*** How criteria do authors plan to use for data saturation?
- ***Table 1: *** Please change “10V15” to “10-15”
- ***Ethical approval***: “ *…a copy of the forms a given to the participant to keep”.* Please change sentence to “a copy of the forms will be given to the participants to keep”
- ***Ethical approval: ***” *All data collected for this study will be kept in a secure password-protected environment….” *Since data collection will take place at two settings, will the data be stored at a central location or at different sites? Also, what kind of platform do authors plan to use to secure sharing of data across sites?

**Conclusions**

- *“….study will advance our understanding of ways health systems….” *I think the focus should extend beyond hypertension management but also diagnosis and control. Also, please avoid using possessive pronouns such as “ours”

Is the study design appropriate for the research question?

Yes

Is the rationale for, and objectives of, the study clearly described?

Yes

Are sufficient details of the methods provided to allow replication by others?

Partly

Are the datasets clearly presented in a useable and accessible format?

Not applicable

Reviewer Expertise:

Cardiovascular health (hypertension), implementation science , qualitative research, mixed-methods, health disparities.

I confirm that I have read this submission and believe that I have an appropriate level of expertise to confirm that it is of an acceptable scientific standard, however I have significant reservations, as outlined above.

# Response

**Published:** 2024-04-08

**Authors:** Brahima Amara Diallo

**Affiliations:** Nutrition and Planetary Health, MRC Unit The Gambia at LSHTM, Banjul, The Gambia

Dear Editorial Board,

 Thank you for allowing us to submit a revised draft of our paper titled “ *Managing hypertension in rural Gambia and Kenya: Protocol for a qualitative study exploring the experiences of patients, health care workers, and decision-makers*”. We appreciate the time and effort that the reviewer has dedicated to providing valuable feedback. We have incorporated changes in the manuscript to reflect most of the suggestions the reviewer has provided.

 Please, find below a point-by-point response to the reviewer’ comments and concerns.

**Reviewer:**

**Summary of the article**

 This study aims to systematically assess the micro (patients/carers), meso (health care workers and facilities) and macro (broader systems) contexts in rural Gambia and Kenya by exploring how people living with hypertension understand and manage their conditions and the perspectives and experiences of those organizing, financing, and delivering hypertension care services. This study is very relevant given the high prevalence of hypertension in sub-Saharan Africa and the potential impact it can have on the population. Understanding ways to enhance hypertension care in SSA is crucial, and this study will offer valuable insights on how to achieve that. While the paper provides valuable insights into the topic at hand, there are areas where improvements can be made to ensure its scientific rigor. Here are some suggestions for enhancing the paper's scientific credibility.

**Authors’ comment:** Thank you for your comment

**Reviewer:**

**Abstract:** ***Background: *** The objective stated here seems to be incomplete. Please ensure that it is the same with the one stated in the introduction section.

**Author’s response: **We acknowledge that the objective provided in the abstract is not as detailed as in the introduction. We are restricted by a word limit, which means that we had to abbreviate the objective in the abstract.

**Reviewer:**

***Methods***: Authors should please provide the methods that they intend to use to assess the capacity and readiness of the health systems.

**Author's response: **This study will not use any standardized assessment tool. We will assess the capacity and readiness of the health systems of both countries based on the accounts from health care providers, decision-makers, and patients by juxtaposing how hypertension should be diagnosed, treated, and controlled as per the national policies and guidelines and what happens in reality in health facilities when health care providers are attending to hypertensive patients, and how the health systems of both countries can be strengthened to offer optimal services for the hypertension care in rural Gambia and Kenya.

**Reviewer:**

***Expected findings: *** Will this study also be able to describe context for hypertension control since the national guidelines and policy documents will be able to provide this information? Authors need to be consistent as to what the focus of their study will be. Will this study address hypertension diagnosis, management and control, or hypertension diagnosis or diagnosis and management?

**Author's response: **As set out in the section “Study aims and objectives”, our overall aim is *to understand the experiences, needs, and practices at the individual, organisational, and system levels to detect, treat, and control hypertension in rural Gambia and Kenya.* We recognize that the terminology is not always clear, with the term ‘management’ taking different meanings in different contexts (e.g., a policy, which would also include primary prevention and a clinical term, that is, reducing blood pressure). Our study specifically focuses on the diagnosis and treatment of hypertension in terms of the processes involved, which ideally lead to an individual’s blood pressure being controlled. Altogether, this can be described as ‘hypertension management’. We have reviewed the text and clarified the terminology, accordingly, using the term ‘management’ throughout. We have added a sentence to the Methods section, clarifying that our understanding of ‘management’ covers diagnosis, treatment, and control, as follows: *For the purposes of this study, we understand ‘management’ to encompass the diagnosis, treatment, and control of hypertension. *(p.7 in the revised manuscript)

**Reviewer:**

**Introduction : **“ *Hypertension is a major cause of premature mortality……an estimated 10.7 million deaths globally”* Please is this estimate annual estimate?

**Authors’ response: **This was an estimate of the burden of hypertension globally from data generated in 2015. We have amended the sentence to read: *Hypertension is a major cause of premature mortality, accounting for an estimated 10.7 million deaths globally in 2015 (1).*

**Reviewer:** “ *Sub-Saharan African (SSA) has among them highest.....” *Please edit this sentence for clarity. I will also suggest breaking this into two sentences to improve clarity.

**Authors’ response:** We apologise for this typological error. We have now revised the sentence to read: *Sub-Saharan Africa (SSA) has the highest burdens, with prevalence rates at around 30%, (2) and in this region, hypertension was implicated in about 1 million deaths in 2019, double the number in 1990 (3).*

**Reviewer:**

**Methods:** *"This is a qualitative study that will use focus group discussion and in-depth interviews with people living with hypertension….” *The participant focus here is different from what was stated in the abstract. Please reconcile both.

**Authors’ response: **Thank you for your observation, we have amended the wording in both the abstract and the methods section accordingly.  The abstract now reads: *We will conduct (i) focus group discussions with people living with hypertension to map a ‘typical’ patient journey through health systems, and (ii) in-depth interviews with patients and family carers, health care workers, decision-makers, and NCD partners to explore their experiences of managing hypertension and assess the capacity and readiness of the health systems to strengthen hypertension management.”*

 The introductory sentence to the methods now reads:

*This is a qualitative study that will use (i) focus group discussion with people living with hypertension and (ii) in-depth interviews with people living with hypertension and their family carers, community health volunteers, health care workers, decision-makers, and NCD partners in The Gambia and Kenya.*

**Reviewer:**

***Patient and public involvement:*** “ *Community members and stakeholders….” *Please provide the list of these stakeholders.

**Authors’ response: **We have clarified the section as follows:

*Community members and stakeholders have been involved in different stages of developing the IHCoR-Africa study project and its implementation. In the Gambia, this included community health volunteers, managers of health facilities, and representatives of the regional health directorate and the Ministry of Health. In Kenya, this included representatives of community advisory groups, community health volunteers, facility managers, county health managers, Ministry of Health representatives, and NCD partners.*

**Reviewer:**

***Conceptual framing*:** “Our overall approach to this study…..” It seems this sentence is incomplete. Authors should please state what they mean by their approach placing patients at the centre. At the centre of what? policy focus?

**Authors’ response: **We agree and have revised the sentence to read: *Our overall approach to this study places people with hypertension (and their family carers) at the centre of the hypertension care pathway.*

**Reviewer:**

***Conceptual framing: …***..” *to improve hypertension management and control in rural Kenya and Gambia” *How about hypertension detection? Authors need to be clear with the focus of their study- Are they interested in improving hypertension diagnosis, management and control, or only management and control? This inconsistency seems to run through the paper.

**Authors’ response: **As noted in our response to an earlier comment, our study focuses on the diagnosis, treatment, and control of hypertension; the term ‘management’ is also used to refer to these in the document. We have reviewed the entire text to ensure consistency in wording.

**Reviewer:**

***Data collection methods:*** Since Kenya and the Gambia operate different healthcare system, how did authors decide the level to choose health care worker participants from in these two countries? I think some statements around this will be helpful to readers.

**Authors’ response: **Thank you for your comment. We believe that we have explained our approach in the subsection “Health care workers”, within the “Data collection” section, where we detail the level of health facilities and types of health care workers and how these will be identified for inclusion in the data collection.

**Reviewer:**

***Data collection methods-IDI:*** *“…as well as exploration of the readiness for and capacity of health facilities….” *Please which level of health facilities are the authors referring TO here, given that there are different levels, and these vary in Kenya and Gambia?

**Authors’ response: **As mentioned in our response to the preceding comment, we provide these details in the subsection “Health care workers” within the “Data collection” section. We describe that we will contact health care workers at different facility levels. Specifically, we note the following (p. 8 of the revised manuscript):

*In The Gambia (Lower River Region), we will invite health care workers involved in hypertension diagnosis and management at village health services, community clinics, and minor health centres (level 1), and the district hospital (level 2). In Kenya (Kilifi), we will consider all levels of health service delivery, including community health services (level 1), dispensaries (level 2), health centres (level 3), as well as sub-county and county hospitals (levels 4 and 5).*

*In both study sites, the selection of facilities to be included in the study will be discussed with the relevant health authorities (Gambia Lower River Regional health authorities and Kilifi County health authorities in Kenya) to identify those with active hypertension management clinics. At each level of health facility, the number of health care workers to include in the data collection will be 1-3.*

**Reviewer:**

***Document Review (objectives 1 and 3):*** *“We will analyse published and unpublished documents including national-level strategy….”* This approach is different from what was stated in the abstract. Please reconcile this.

**Authors’ response: **Thank you for raising this point. The information provided in the abstract had, of necessity, to be shortened in line with the word limit requirement of 300 words. We have not changed the text in the abstract for that reason.

**Reviewer:**

***Document Review (objectives 1 and 3)***: “ *We will also conduct Google scholar searches…” *Please what will the search term focus on?

**Authors’ response: **We have amended the sentence to read: *We will also conduct Google Scholar searches to identify any additional documents of relevance to the study aim, using the following search terms “guideline/s or protocol”, “hypertension”, “diagnosis”, “treatment”, “control” and “sub-Sahara/n Africa”.*

**Reviewer:**

***Document Review (objectives 1 and 3):*** *“…and The Gambia to collate clinical protocols and standard operating procedures as they relate to hypertension control”* In sub-Saharan Africa, standard operating procedures are usually not written down especially in lower-tier facilities. In such situation, how will the authors obtain the clinical protocols and standard operating procedures?

**Authors’ response: **Thank you for this important observation. We agree and have amended the sentence to read: *Furthermore, we will approach individual health facilities in the study locations in Kenya and The Gambia to collate clinical protocols and standard operating procedures relating to the management of hypertension where these are available.*

**Reviewer:**

***Data collection/health care workers: *** “ *In the Gambia (Lower River Region), we will invite health care workers involved in hypertension….” *Authors should please provide the number of participants that they intend to recruit from each of these levels of care.

**Authors’ response: **We have added the following sentence: *At each level of health facility, the number of health care workers to include in the data collection will be 1-3 individuals.*

**Reviewer:**

***FGD and interview process:*** How criteria do authors plan to use for data saturation?

**Authors’ response: **We do not plan to use a formal numerical approach to determine data saturation (see e.g. Hennink and Kaiser 2022). We have added the following sentences to the “Health care workers” subsection within the “Data collection” section (p. 9 in the revised manuscript): *Data saturation will be assessed based on whether the study team is not able to identify any new themes after conducting a certain number of consecutive interviews in each category of informants in each study setting. This means that recruitment will stop when data that has been collected is viewed as adequate in relation to the study objectives and that any further data collection will not generate any additional insights* *.*

**Reviewer:**

***Table 1: *** Please change “10V15” to “10-15”

**Authors’ response: **We have corrected the table.

**Reviewer:**

***Ethical approval***: “ *…a copy of the forms a given to the participant to keep”.* Please change sentence to “A copy of the forms will be given to the participants to keep”

**Authors’ response: **We have corrected the sentence to read: *A copy of the forms will be given to the participant to keep, …*

**Reviewer:**

***Ethical approval: ***” *All data collected for this study will be kept in a secure password-protected environment….” *Since data collection will take place at two settings, will the data be stored at a central location or at different sites? Also, what kind of platform do authors plan to use to secure sharing of data across sites?

**Authors’ response: **We have amended the paragraph to read as follows: *Data collected in each study country will be kept in a secure password-protected environment and any hard copies of interview transcripts and consent forms will be kept in a locked cabinet. Once data have been processed and databases created, they will be encrypted, password-protected and saved on a secured server under each institutional data protection policies, i.e. MRC Gambia (MRCG) and KEMRI-Wellcome Trust Research Programme (KWTRP).* *Encrypted data from both study sites excluding any personal identifiers will later be transferred to a secure server at LSHTM to be used by researchers across different sites for cross-country analysis.*

**Reviewer:**

**Conclusions:** *“….study will advance our understanding of ways health systems….” *I think the focus should extend beyond hypertension management but also diagnosis and control. Also, please avoid using possessive pronouns such as “ours”

**Authors’ response: **we have amended the sentence to read: *“ Using a pragmatic and comprehensive methodological approach, this study will provide insights into ways health systems in two countries are ready for change to improve hypertension management.*”
